# Eyelash Trichomegaly: Unusual Feature Associated with Systemic Lupus Erythematosus Patient

**DOI:** 10.1155/2022/1320992

**Published:** 2022-08-04

**Authors:** Amjad M. AlWarawreh, Ahmad M. AlWarawreh, Bashar F. Jarrar, Asem H. Aldebei, Mahasen S. Ajarmeh, Fadi F. Ayyash, Manal A. Abu Al-Ghanam

**Affiliations:** Royal Medical Services (RMS), Amman, Jordan

## Abstract

Eyelash trichomegaly is a rare disorder in which normal features of eyelashes such as length, color, thickness, or curling changes. It may occur due to many causes, such as the presence of other disorders such as HIV infection, congenital anomalies like oculocutaneous albinism, or Oliver–McFarlane syndrome. It may be linked to the use of certain drugs and can also be present as an isolated trait by birth. Here, we report a rare case of eyelash trichomegaly in a 19-year-old female having diffused alopecia, diagnosed with systemic lupus erythematosus.

## 1. Introduction

Eyelash trichomegaly is defined as a condition in which eyelashes exceed their normal length (12 mm or more), curling, pigmentation, or thickness [1, 2].

Gray, in 1944, for the first time, used the term “trichomegaly” while observing a lymphoma patient with abnormally long eyelashes [3]. This disease may occur as a clinical feature associated with other acquired or congenital diseases. The common congenital anomalies associated with this condition include oculocutaneous albinism or Oliver–McFarlane syndrome and Cornelia de Lange syndrome, while the most common acquired conditions to which it is linked include HIV infection, alopecia areata, diseases affecting connective tissues like dermatomyositis and systemic lupus erythematosus. Certain medications such as cyclosporines and topical latanoprost can also trigger eyelash trichomegaly. The exact mechanism by which it develops has not been extensively studied. A summary of the conditions which may trigger trichomegaly is given in Table 1.

Here, we present a case of eyelash trichomegaly associated with systemic lupus erythematosus. Hair loss in a diffused pattern is also present in the patient. All information presented here were obtained with the permission of the patient's parents.

## 2. Case Report

**Figure 1:**
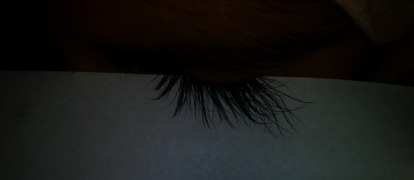
Eyelash trichomegaly in SLE patient.

For nine months, a Jordanian girl, 19 years old (Figure 1), had typical symptoms such as weakness, malaise, joint discomfort, no or diminished appetite, sensitivity to sunlight, and low-grade fever. Since last year, she has seen hair loss as well as an increase in the length and curl of her eyelashes.

**Figure 2:**
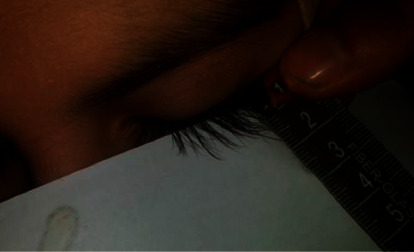
Eyelash trichomegaly in SLE patient.

Her medical record for the last six months showed that she had taken no medicine except prednisolone orally (20 mg/day). Her family history shows no cases of hypertrichosis. Mild signs and symptoms such as erythema in the paranasal region and diffused hair loss in the scalp were discovered during the examination (classified as type-1 according to Ebling and Rook's five-stage classification of hair loss pattern for females). Trichomegaly was observed as her eyelashes measured 17 mm long (Figure 2).

Hb 9.8 g/dL, TLC 2800/mm^3^, ESR 72 mm/h, and the number of platelets present were 105,000/mm^3^ indicative of thrombocytopenia, which were all found on her hematological reports. No proteins or casts were seen in the urine examination. Antinuclear antibodies (1 : 320, homogeneous pattern) and anti-dsDNA antibodies were detected. The patient was diagnosed with SLE and was directed to consult a rheumatologist for treatment.

## 3. Discussion

During development at embryonic stages, eyelashes are the first hair to grow. They appear roughly around the 12^th^ week of gestation. Their growth completes in a cycle of three stages in six months: anagen phase (30 days), catagen phase (15 days), and telogen phase (over 100 days).

The normal length of eyelashes of the eyelid is 8–12 mm. An increase of this length or thickening, curling, and pigmentation is known to be eyelash trichomegaly [1, 2]. It may be present at birth in association with other congenital syndromes, which include Cornelia de Lange syndrome and Oliver–McFarlane syndrome (most common), Hermansky–Pudlak syndrome, and congenital heart disease (uncommon) and Aghaei–Dastgheib syndrome, cone-rod syndrome, Goldstein–Hutt syndrome, and phylloid hypomelanosis (rare).

It may also show up as an isolated benign family feature in rare cases. These changes also develop as a characteristic feature of other syndromes developed later in life, such as atopic dermatitis, uveitis, vernal keratoconjunctivitis, and HIV infection. In rare cases, it may also be associated with other acquired syndromes such as alopecia areata, metastatic renal adenocarcinoma, dermatomyositis, and systemic lupus erythematosus.

Certain drugs are also key players in the development of these changes. The drugs to which this condition is linked most commonly include cyclosporine, inhibitors of epidermal growth factor receptor (EGFR), cetuximab, and frequent use of prostaglandin analogs such as latanoprost and travoprost [2, 4] (Table 1).

SLE is a chronic autoimmune disease involving multiple systems. The autoantibodies which are produced cause a wide spectrum of medical complications. Hair growth patterns change as a result of SLE. In the classification criteria, nonscarring alopecia has also been included [5]. Diffuse alopecia affects up to 50% of patients, and uncontrolled, short, broken-off “lupus hair,” which affects 30% of patients [6].

The pathophysiology of eyelash trichomegaly has been inadequately explored because of a lack of histology and organ culture data for eyelash hair. A molecular mechanism for trichomegaly development has been proposed. According to this theory, stem cell inactivation in different regions of the hair follicle by the nuclear factor of activated T cells (NFAT) may lead to eyelash trichomegaly. EGFR regulates the growth cycle of eyelash hairs, and its inhibition may lead to trichomegaly [7].

In this report, we document a case of trichomegaly associated with SLE along with diffused hair loss. The development of eyelash trichomegaly in our patient may have been caused by EGFR inhibition.

## Figures and Tables

**Table 1:** Eyelash trichomegaly: causes and occurrence.

Conditions	Common	Uncommon	Rare
Congenital	Cornelia de Lange syndrome Oliver–McFarlane syndrome	Congenital heart disorders Familial trichomegaly Hermansky–Pudlak syndrome	Aghaei–Dastgheib syndrome Cone-rod dystrophy Goldstein–Hutt syndrome
Acquired	Atopic dermatitis HIV Uveitis Vernal keratoconjunctivitis		Alopecia areata Cancer Dermatomtositis Systemic lupus erythematosus
Drugs	EGFR inhibitors Monoclonal antibodies Tyrosine-kinase inhibitors	Panitumumab Interferon-alpha	Cyclosporine Tacrolimus Topiramate Zidovudine

## Data Availability

The data used to support the findings of this study are included within the article.
